# Vectorgastrogram: dynamic trajectory and recurrence quantification analysis to assess slow wave vector movement in healthy subjects

**DOI:** 10.1007/s13246-024-01396-y

**Published:** 2024-03-04

**Authors:** Gema Prats-Boluda, Jose L. Martinez-de-Juan, Felix Nieto-del-Amor, María Termenon, Cristina Varón, Yiyao Ye-Lin

**Affiliations:** Centro de Investigación e Innovación en Bioingeniería (CI2B), Universitat Politècnica de València (UPV), Valencia, Spain

**Keywords:** Electrogastrography, EGG, Vectorgastrogram, RQA, Recurrence plot

## Abstract

Functional gastric disorders entail chronic or recurrent symptoms, high prevalence and a significant financial burden. These disorders do not always involve structural abnormalities and since they cannot be diagnosed by routine procedures, electrogastrography (EGG) has been proposed as a diagnostic alternative. However, the method still has not been transferred to clinical practice due to the difficulty of identifying gastric activity because of the low-frequency interference caused by skin–electrode contact potential in obtaining spatiotemporal information by simple procedures. This work attempted to robustly identify the gastric slow wave (SW) main components by applying multivariate variational mode decomposition (MVMD) to the multichannel EGG. Another aim was to obtain the 2D SW vectorgastrogram VGG_SW_ from 4 electrodes perpendicularly arranged in a T-shape and analyse its dynamic trajectory and recurrence quantification (RQA) to assess slow wave vector movement in healthy subjects. The results revealed that MVMD can reliably identify the gastric SW, with detection rates over 91% in fasting postprandial subjects and a frequency instability of less than 5.3%, statistically increasing its amplitude and frequency after ingestion. The VGG_SW_ dynamic trajectory showed a statistically higher predominance of vertical displacement after ingestion. RQA metrics (recurrence ratio, average length, entropy, and trapping time) showed a postprandial statistical increase, suggesting that gastric SW became more intense and coordinated with a less complex VGG_SW_ and higher periodicity. The results support the VGG_SW_ as a simple technique that can provide relevant information on the “global” spatial pattern of gastric slow wave propagation that could help diagnose gastric pathologies.

## Introduction

Functional gastrointestinal disorders are highly prevalent in the population and in primary and secondary care centers. Approximately 11% of the population suffers from a chronic digestive disease, with a prevalence rate as high as 35% for those over 65 years of age [1]. A recent large-scale multinational study found them to be present in more than 40% of the world’s population [2]. Gastrointestinal diseases are responsible for more than 36.8 million outpatient visits and more than 3.8 million admissions, with 403,699 readmissions, in the USA annually [3]. Gastrointestinal disorders and cancers were also associated with 255,407 deaths in the USA in 2018 [3]. In addition to negatively impacting the patients’ quality of life, they also involve a heavy economic burden which reached $119.6 billion in this country in 2018 [3].

Many routine medical tests such as endoscopic exams, CT scans, blood tests, and radiological imaging fail to detect functional gastrointestinal motility disorders, given that there is no inflammatory, infection, or structural abnormality [4]. While gastric emptying scintigraphy (GES) utilizing radio-labeled meals is widely regarded as the benchmark for assessing patients with gastrointestinal motility disorders, concerns about its reliability have been raised by gastroenterologists because results may be influenced by factors such as age, gender, and the type of food ingested during the test [5]. The challenge stems from inconsistent results attributed to the absence of a standardized procedure regarding the meal type, patient positioning, image acquisition frequency and duration, and quantitation method. The lack of uniformity in these aspects impacts the establishment of reported normal values and, consequently, hinders the comparison of results across different institutions. These divergent test outcomes further complicate the decision-making process for patient treatment [6]. Attempts to solve this issue by defining standardized protocols have been carried out [7]. Nonetheless, the interpretation of these test results may be challenging, and it is essential to consider other clinical symptoms and diagnostic information in conjunction with test findings.

Electrogastrography (EGG) has is an alternative diagnostic technique for this type of dysfunction. EGG is the recording of gastric electric activity on the upper abdominal surface and it is compounded by slow waves (SW) and spike bursts [8]. Slow waves are pacemaker activity generated and propagated through the network of interstitial cells of Cajal’s [9]. Spike bursts are rapid action potentials that only appear on the slow wave plateau during stomach contractions, and their energy is directly linked to the intensity of gastric contractions [9]. In healthy humans, the gastric SW dominant frequency is typically around 3 cycles per minute, originates in the upper corpus region and propagates towards the pylorus [10]. Normal EGG recordings have traditionally been defined with a dominant frequency in the range of 2–4 cpm for at least 70% of the recording time [11, 12]. Bradygastria and tachygastria are defined as SW dominant frequency within 0.5–2 cpm and 4–9 cpm, respectively. The absence of a dominant peak in the SW power spectrum is considered gastric arrhythmia [13], even though it does not represent gastric motility but regulates the rhythm of the maximum stomach contractions [14]. Previous studies have shown that an impaired gastric slow wave is linked to several gastrointestinal motility disorders including unexplained chronic nausea and vomiting, gastroparesis, functional dyspepsia, reflux with regurgitation and motion sickness [15–19].

Despite its promising results, the clinical application of the EGG technique is still limited, due to ongoing controversy regarding the association between EGG temporal and spectral parameters and gastric pathologies [20, 21]. Using single-channel temporal and spectral parameters, Leahy found that the EGG’s diagnostic value relies on its high specificity (~ 93%), and only 36% of functional dyspepsia patients showed an abnormal EGG [22]. No correlation was found between the symptoms and the traditional temporal and spectral EGG parameters in subjects with functional dyspepsia and gastroparesis [21]. While an abnormal EGG usually involves delayed gastric emptying, a normal EGG does not guarantee normal gastric emptying (sensitivity < 50%) [20]. In contrast, high-resolution gastrointestinal serosal electrical mapping suggested that slow wave propagation may constitute reliable signatures of the occurrence of dysrhythmic events [15, 16, 23–25]. Multiple studies have sought to determine slow wave propagation biomarkers through non-invasive body surface gastric potential mapping (BSGM or HR-EGG) [11, 21, 26]. Gharibans et al. proposed HR-EGG mapping from an array of 5 × 5 cutaneous electrodes to non-invasively estimate the gastric slow-wave direction and speed [11]. They successfully estimated the slow wave’ propagation direction (181 ± 29°) and speed (3.7 ± 0.5 mm/s), which was consistent with serosal recording of slow-waves: corpus 3 mm/s in the corpus and 5.7 mm/s in the antrum for healthy subjects [10, 27]. However, this type of estimation involves a cumbersome and time-consuming recording protocol that requires a 5 × 5 electrode array connected to bioamplifiers, a process with limitations to carry out in clinical practice although advanced signal processing techniques would yield promising results for clinical setting from HR-EGG.

Gastric vector displacement or vectorgastrography (VGG) analysis can also be used to assess the propagation events that move through the entire organ. The VGG was conceived by Martin and Thillier and consisted of a trapezium configuration with the angular points where the extremities joined the trunk [27]. Similar to the well-known vectorcardiogram, the VGG is the spatial representation of electromotive forces generated by gastric activity. It can be interpreted as a variation of the instantaneous gastric vector displacement viewed from a relatively long distance [28]. The electrical activity generally depends on the geometric relationship between bioelectric dipole intensities and the spatial orientation of the electrical dipoles, which vary during the bioelectric activity cycle, forming loops that connect both ends of the vector. The degree of organization of the bioelectric events is also reflected in gastric vector displacement loops, i.e. the more organized events show more self-similarity and smoother vector representation than the 12-lead electrocardiogram. The vectorcardiogram (VGG) has been shown to provide enhanced specificity and sensitivity in diagnosing ischemia, hypertrophy, chamber enlargement, fascicular blocks and ventricular pre-excitation in association with myocardial infarction [28–32], so that VGG analysis could provide another previously unconsidered potential indicator of gastric dysfunction. However, its ability to study gastric vector path dynamic behavior remains unclear, since no further studies have been conducted in the field.

Cardiac vector loop characterization usually includes morphological features such as Qvector, Rvector, Svector magnitude [33] and different (VGG) octant features including position, average, maximum, ratio and variance of Rvector, Tvector and Qvector [33], QRS area [34] and dynamic properties [35] such as speed, curvature and phase angle. The recurrence plot arose as an advanced nonlinear method and is widely used to characterize dynamic VGG behavior [36–39]. The recurrence plot is a graphical tool based on phase space reconstruction that analyses the dynamic behaviour of complex systems [40], in which recurrence is the ability to return to the same state on the phase space after a certain period of time. Recurrence plots can be used to determine both specific large- and small-scale patterns, hidden patterns and structural changes in data [40]. The idea is to use phase space reconstruction to extract valuable features by extending an M-dimensional time series to a high dimensional phase space, and characterize this latter through 2D representation of its recurrence [41]. Although they only allow qualitative analysis of dynamic behaviour with no quantitative measure. Recurrence quantification analysis (RQA) was introduced to overcome this drawback and has obtained promising results in different applications, including biomedical signal characterization [36–39, 42]. RQA is a statistical quantification of the recurrence plot that consists of quantifying the diagonal and vertical/horizontal lines to assess laminar, divergent or nonlinear transition behavior [43] and has been helpful in detecting phase transition, dynamic regime characterization and synchronization analysis, even for short, non-stationarity and noisy data [40].

The aim of this work was thus to determine the feasibility of obtaining the gastric slow wave vectorgastrogram VGG_SW_ by studying this gastric vector loop’s behaviour and quantifying its dynamic properties in the fasting and postprandial states of healthy subjects. We specifically worked out the 2D VGG_SW_ global displacement length and its spatial orientation and also analysed the VGG_SW_ recurrence statistics using RQA analysis.

## Materials and methods

### Signal acquisition

A total of 18 recording sessions were conducted on healthy young subjects (10 men and 8 women) with a body mass index of 21.8 ± 2.7 kg/m^2^ and aged between 20 and 24 years old. They were previously informed about the nature of the study and provided written informed consent forms. The Universitat Politècnica de València ethics committee approved the study protocol (ref. P02_17_06_21), which adhered to the Declaration of Helsinki.

**Fig. 1:**
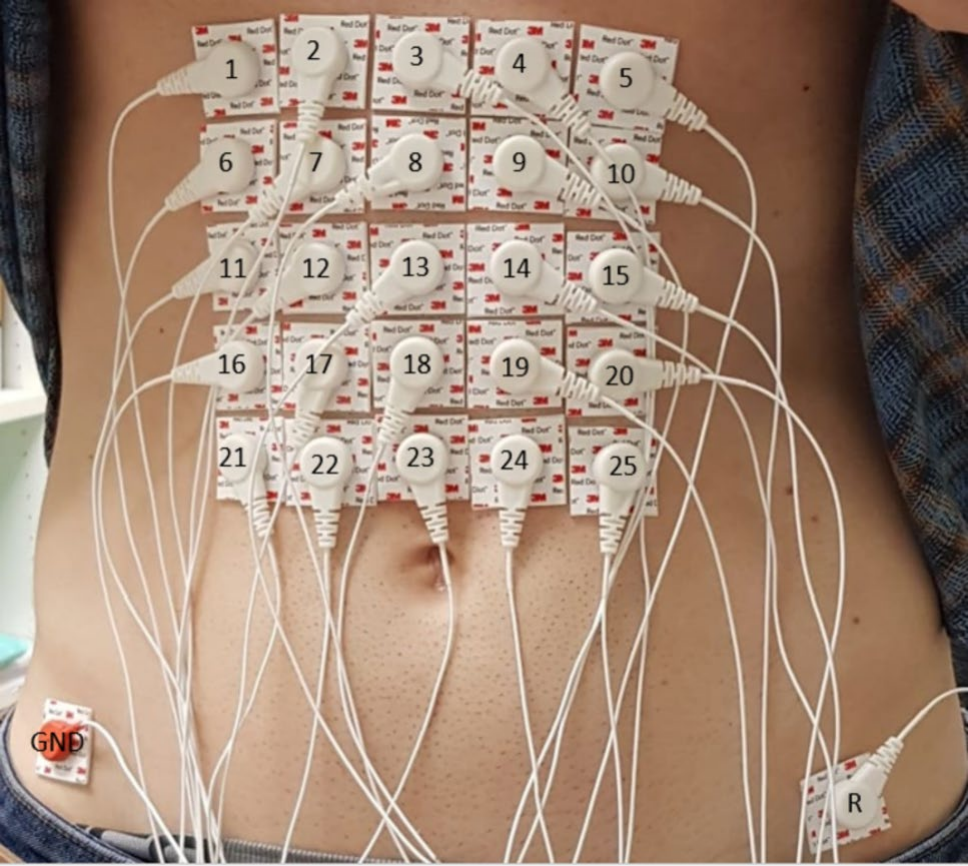
Electrode positioning for EGG signal recording

The sessions included 30-min of recording in a fasting state (> 8 h) and 30-min of recording after ingesting a solid meal (400 kcal with a fat content of 18% and 0.25 L of water). The abdominal skin was carefully prepared before the recording sessions by an abrasive paste to reduce skin–electrode contact impedance and cleaned with alcohol. A 5 × 5 electrode matrix of disposable Ag/AgCl electrodes (Red Dot 2660–5, 3M, St. Paul, MN, USA) with an interelectrode distance of 2.5 cm was positioned on the upper abdominal surface, as shown in Fig. 1). Electrode Nº 3 (top row middle column) was placed just below the apophysis xiphoid. The reference (R) and ground electrodes (GND) were placed on the left and the right hips, respectively (see Fig. 1). The 25 monopolar bioelectrical signals were acquired by the SAGA 32 + bioamplifier (TMSi) at a sample rate of 500 Hz. Taking into account the characteristics of gastric slow wave (Healthy: 2–4 cpm, bradigastria 0.6–2 cpm and tachygastria: 4–9 cpm), we bandpass filtered EGG signals at [0.6, 12] cpm by a zero phase Butterworth order 5 filter, and decimated to a 4 Hz sample rate, which is a commonly used for analysing the gastric SW component [8].

### Gastric slow wave identification

#### Multivariate variational mode decomposition (MVMD)

Monopolar electrogastrographic recordings are susceptible to picking up interference, such as low-frequency interference associated with the contact potential between the electrodes and the skin, whose spectrum is very close to the gastric SW bandwidth, if not overlapping, which makes it extremely difficult to identify the gastric SW. In this context, standard FFT was ineffective in identifying slow wave frequency components in noisy signals due to the limited frequency resolution and its side lobes. In this work, we propose the multivariate variational mode decomposition (MVMD) technique to robustly identify the gastric slow wave component. Afterward, autoregressive model, an alternative to Fourier transform for high-resolution spectral estimation of a short time series, was used to determine gastric slow wave frequency. Indeed, AR model was commonly used for gastric slow wave frequency identification in previous studies [44–47].

MVMD is an extension for multichannel signals of the variational mode decomposition, which is quite useful in non-stationary data [48, 49]. This method identifies a set of common multivariate modulated oscillations in the input data with a minimum collective bandwidth that can reconstruct input data channels. The Hilbert transform-based canonical analytic signal representation modeled the multivariate oscillations that must be common between the channels as the multivariate instantaneous frequency [49].

The objective of MVMD is to extract a predefined number k of modes from an input signal with C channels. The modes are defined as vectors of amplitude and frequency modulated (AM-FM) [50] that have to meet two conditions: (a) The sum of the extracted modes must exactly recover the original signal; (b) The sum of the bandwidths of the extracted modes must be minimal. Signal decomposition thus consists of obtaining the k modes that minimize the function of cost and the sum of the bandwidths to maintain the signal reconstruction. In this work the number of extracted modes was fixed at k = 10, and the penalty term, α = 2000 [51]. After applying MVMD in the fasting and fed recordings, the mode with a dominant frequency between 2 and 4 cpm was identified as SW fundamental gastric component. The SWF was then obtained as the dominant frequency of the identified mode computed in each 5-min window through a covariance parametric autoregressive AR model order 120 in all the windows and channels.

To confirm the MVMD performance technique, the SWF was worked out from the raw conditioned EGG records without applying the MVMD, following the same method described in the previous paragraph.

#### Cross spectrum

A global gastric SW frequency (GSWF) was calculated from all the recording signals. The conditioned EGG signals were segmented in 5-min analysis windows with an 80% overlap. The cross-spectrum using the Welch periodogram was calculated for all channel pairs in the 5-min analysis windows with an 80% overlap, NFFT equal to 8192 and being the sampling frequency, fs = 4 Hz with a corresponding definition in frequency (fd) of 0.029 cpm ≈ 0.03 cpm. We then determined each cross-spectrum’s dominant frequency in the typical SW frequency range from 2 to 4 cpm. The GSWF was finally obtained as the mode value of the different dominant frequencies between 2 and 4 cpm.

#### Gastric SW features

To assess both the raw signals and mode signal extracted from the MVMD and their effectiveness in detecting the gastric SW and also to characterise the gastric SW in the fasting and fed states, we computed the following fasting and fed indicators per channel:

- Detection rate, DFCS (%): the ratio between the number of 5-min windows with a dominant SWF within the range of GSWF ± 0.3 cpm and the total number of windows analysed and channels [46].
- Frequency instability coefficient, FIC (%): the ratio between the standard deviation and the average value of the SWF, which represents gastric SW variability throughout the recording [46, 52, 53]. Lower values indicate higher stability of frequency components over time.
- Slow wave frequency, SWF (cpm): the mean frequency obtained from the 5-min windows in which the slow frequency was detected (the range of GSWF ± 0.3 cpm).
- Signal amplitude RMS (μV): the root mean square value of the preprocessed surface signals bandpass filtered between 0.6 and 12 cpm.

Average values for DFCS, FIC(%), SWF and RMS for all channels were obtained. Wilcoxon signed-rank tests were carried out to assess the performance of the MVMD in detecting gastric SW compared to the traditional cross-spectrum techniques, and to analyse the gastric SW differences in both the fasting and fed stages. Statistically significant differences were considered when p < 0.05 (Wilcoxon-Mann–Whitney paired test). Furthermore, we calculated the absolute value of the Cliff’s delta effect size to assess the magnitude of the effect. The p value signifies the presence of the effect, while Cliff’s delta effect size provides information about both its magnitude and direction [54].

### Slow wave vectorgastrogram (VGG_SW_)

In this work we focused on assessing the 2D vectorgastrogram in the X–Y (horizontal-vertical) plane (coronal plane in anatomical terms) of the bipolar signals without considering the Z axis, for simplicity. We obtained the vectorgastrogram associated with the fundamental component of the gastric slow wave, VGG_SW_, previously identified by the MVMD. The results were those of the bipolar combination of gastric SW modes from channels 1 and 4 (X-axis, channel 4 minus channel 1) and those from channels 3 and 18 (Y-axis channel 3 minus channel 18), since it was intended to analyze the direction of propagation of the vector associated with the gastric slow wave propagation. Since vectorgastrogram assesses changes in instantaneous gastric vector displacement viewed from a longer distance, associated with propagated events rather than local activity, we set a minimum inter-electrode distance of 7.5 cm. Then we tested different combinations of bipolar pairs of signals for configuring the X–Y axis, obtaining similar or slightly worse results than those corresponding to the selected axis. Therefore, the average of more vertical/horizontal pairs would obtain similar but not better results [52].

#### Trajectory dynamics

Five parameters were defined to characterise the VGG_SW_ fasting and fed dynamic trajectory based on the characterisation of vectorcardiographic trajectories [35, 55]:

These parameters could serve as biomarkers to assess gastric conditions.

- R1 (n.u.) is the relationship between the sum of differential absolute values of the vertical bipolar signal (Yi) and the sum of differential absolute values of the horizontal bipolar signal (Xi). It assesses the verticality vs horizontality of the displacement of the gastric vector along the trajectory of the VGGSW associated with the 30-min recording.
  1 $${\text{R}}1 = \frac{{\mathop \sum \nolimits_{{{\text{i}} = 1}}^{{\text{N}}} \left| {{\text{Y}}_{{\text{i}}} - {\text{Y}}_{{{\text{i}} - 1}} } \right|}}{{\mathop \sum \nolimits_{{{\text{i}} = 1}}^{{\text{N}}} \left| {{\text{X}}_{{\text{i}}} - {\text{X}}_{{{\text{i}} - 1}} } \right|}}$$
  where N is the number of data of the VGG_SW_.
- R2 (n.u.) is the sum of the relationship between the differential absolute values of the vertical bipolar signal (Yi) and the horizontal bipolar signal (Xi), divided by the number of data values (N). It assesses whether there is an average predominance of verticality horizontality in the VGG_SW_. displacement
  2 $${\text{R}}2 = \frac{1}{{\text{N}}}\cdot\mathop \sum \limits_{{{\text{i}} = 1}}^{{\text{N}}} \frac{{\left| {{\text{Y}}_{{\text{i}}} - {\text{Y}}_{{{\text{i}} - 1}} } \right|}}{{\left| {{\text{X}}_{{\text{i}}} - {\text{X}}_{{{\text{i}} - 1}} } \right|}}$$
- Rv2 (n.u.) is the relationship of the absolute values between vertical velocity and horizontal velocity at maximum velocity modulus. This is a simplified way of estimating the maximum slope of the VGG_SW_. Trajectory [35].
  3 $${\text{Rv}}2 = \frac{{\left| {{\text{Y}}_{{\text{m}}} - {\text{Y}}_{{{\text{m}} - 1}} } \right|}}{{\left| {{\text{X}}_{{\text{m}}} - {\text{X}}_{{{\text{m}} - 1}} } \right|}}$$
  where m is the index for the maximum value of the velocity modulus (vi):
  4 $$\left| {{\text{v}}_{{\text{i}}} } \right| = \frac{{\sqrt {\left( {{\text{X}}_{{\text{i}}} - {\text{X}}_{{{\text{i}} - 1}} } \right)^{2} + \left( {{\text{Y}}_{{\text{i}}} - {\text{Y}}_{{{\text{i}} - 1}} } \right)^{2} } }}{{{\text{t}}_{{\text{s}}} }}$$
  where ts corresponds to the sampling time, the inverse of the sample rate, 1/fs = 1/4 = 0.25 s
- Angle (degrees) is the angle value of the major axis of the VGGSW with respect to the X axis (1–4 electrode). The major axis was identified by principal component analysis (PCA).
- AXR (n.u.) is the ratio between the major and minor axes, identified by principal component analysis (PCA).

#### Recurrence quantification analysis (RQA)

RQA is a nonlinear analysis method that quantifies the number and duration of the recurrences in a dynamic system characterised by its spatial trajectory, which is the VGG_SW_ previously described in the present study (3.3). A recurrence state at the time i and at different time j is plotted as a square matrix (R(i,j)) with the two axes representing time; i.e. [40]:

5 $${\text{R}}\left( {{\text{i}},{\text{j}}} \right) = {\text{H}}\left( {{\upvarepsilon } - { }\left\| {{\text{VGG}}_{{{\text{SW}}}} \left( {\text{j}} \right) - {\text{VGG}}_{{{\text{SW}}}} \left( {\text{i}} \right)} \right\|{ }} \right),\;\;\;\;\;\;\;\;\;\;\;\;{\text{i}},{\text{j}} = 1,2, \ldots \ldots \ldots \ldots ,{\text{N}}$$

where H() is the Heaviside function, ε is a specified threshold distance, $\left\| \cdot \right\|$ the Euclidean distance norm and N the number of states (N = 7200 corresponds to 30 min).

To characterise the recurrence matrixes, a threshold was calculated as a constant (th) multiplied by the median of all the distances in the matrix, which can be considered as the distance threshold below which two points are considered recurrent. A grid search for “th” was assessed (0.2, 0.3, 0.4 and 0.5). Since the aim is to find the number of diagonal and vertical lines that exceed the minimum length (tmin), a scan was carried out of minimum lengths of 2, 4, 6 and 8 points. The best results for differentiating between fast and fed stages were obtained for th = 0.4 and tmin = 6 points (1.5 s).

Notably, diagonal line length structures in the recurrence matrix are associated with similar local time evolution in different parts of the trajectory, thus indicating a high predictability and a deterministic system [43]. The dynamic system generates a vertical line in the recurrence plot when it remains in the same state for some time, i.e. the vertical line length is associated with system stability [43]. The following RQA metrics were worked out to quantify the predictability, stability and complexity of the fast and fed VGG_SW_ recurrence matrices:

- The recurrence ratio RR (%) is the percentage of the number of recurrent dots below the threshold over the total of dots in the recurrence plot. High RR(%) values mean high repeatability and predictability.
- Determinism DET (%) is the percentage of recurrent dots that form diagonal lines (parallel to the main diagonal or identity line), over the total dots in the recurrence plot. The presence of these lines reveals a deterministic structure, i.e. high DET values exist in periodic systems, in which the disappearance of periodicity causes the fragmentation of diagonal lines.
- The average length of diagonal lines, LMEAN, is the average of the diagonal lines that exceed tmin = 6 points (1.5 s). It is related to the divergence of the trajectories, since it indicates how long the trajectory is maintained. LMEAN is normalized to the time of a signal cycle (GSWF) to avoid the bias that an increase in frequency could introduce. The higher the LMEAN values, the higher the predictability of the dynamic system.
- Entropy ENT is the Shannon entropy of the diagonal line distribution, which represents the complexity of the deterministic structure of the system [40]. High ENT values from a recurrence plot correspond to lower chaos and complexity of the signal [56–58].
- Trapping time TT is the average length of the vertical lines that exceed tmin = 6 points (1.5 s) and indicates how long the system remains in a particular state. TT is also normalised to the time of a signal cycle (GSWF) to reduce the effect that a change in frequency could introduce.

Wilcoxon signed-rank tests were computed to determine whether there were statistically significant differences (p < 0.05) in VGG_SW_. trajectory dynamics metrics and those resulting from in fast and fed VGG_SW_. recurrence matrices. Additionally, we calculated Cliff’s delta effect size to assess the magnitude of the effect.

## Results

### Traditional gastric slow wave features

**Fig. 2:**
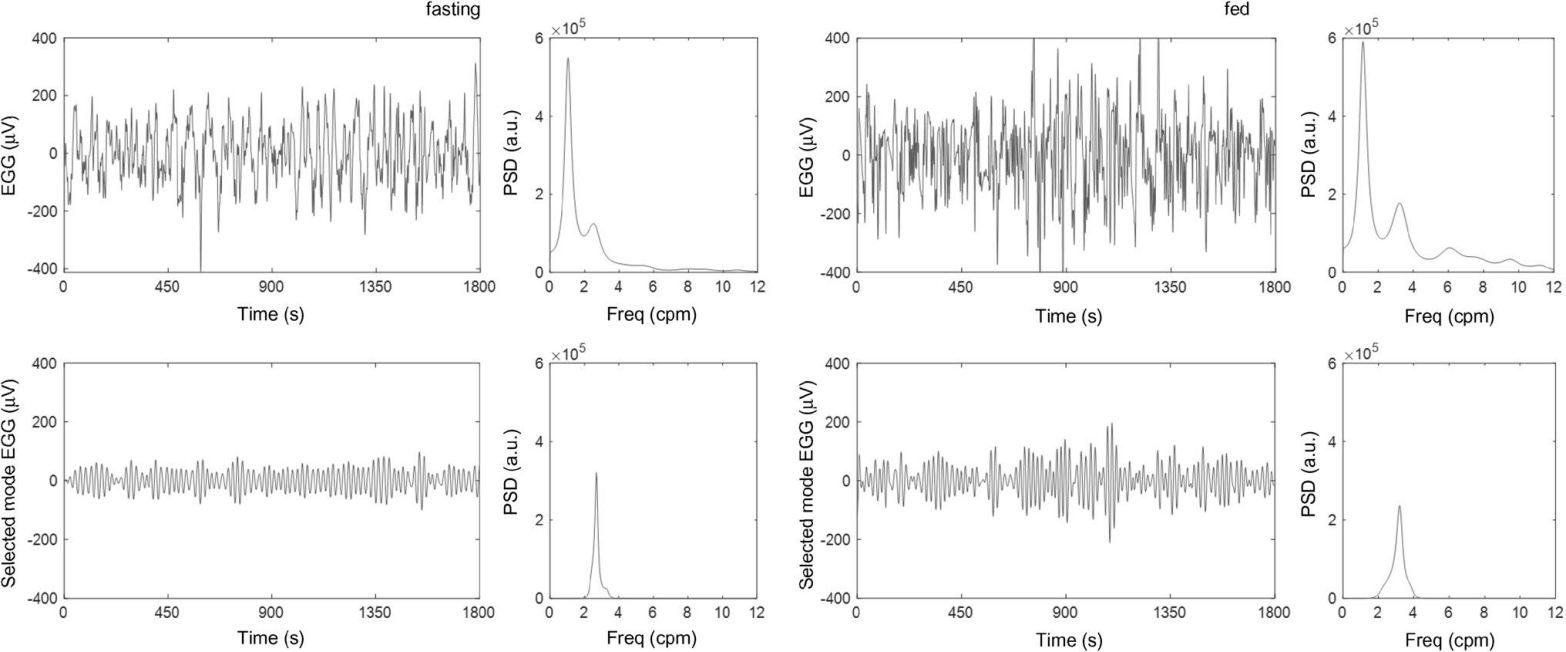
Comparison between raw EGG signals (upper graphs) and mode functions related to the main component of the gastric slow wave from MVMD signals (lower graphs). Each signal is displayed with its PSD on the right side. The signals on the left are from fasting (subject 1) and the signals on the right are from fed (subject 8)

Figure 2 shows the result of applying MVMD to improve the identification of the main component of the gastric SW. The recorded EGG raw signals are shown in the upper graphs, together with the PSDs (AR model order 120) to their right. It is observed that the gastric SW, which is between 2 and 4 cpm, is masked by low-frequency interference. After running the MVMD technique, mode functions that detect a peak in PSD between 2 and 4 cpm are selected, and they can be observed in lower graphs.

**Fig. 3:**
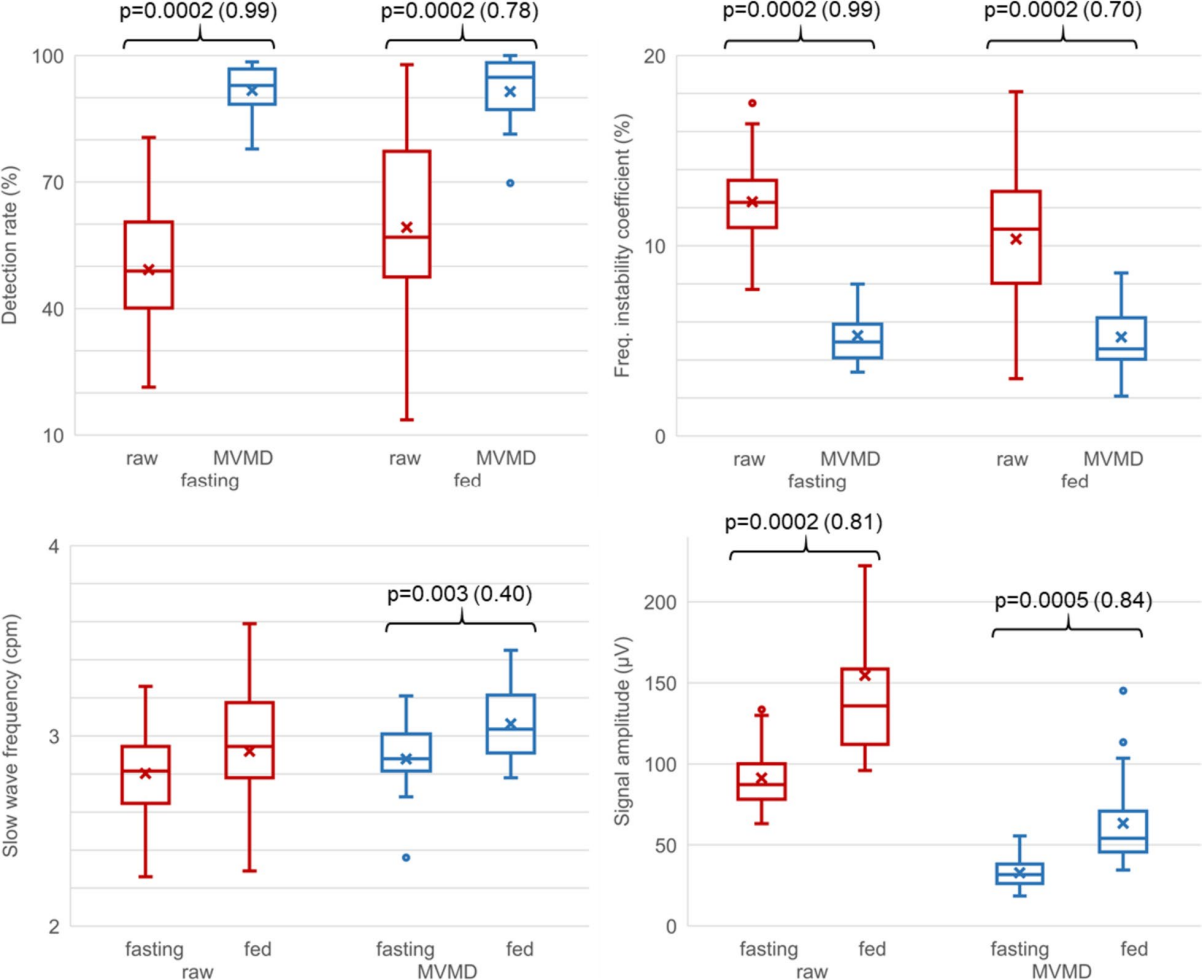
Box and whisker plots of the gastric slow wave identification parameters without MVMD (red traces) and with MVMD (blue traces). The upper graphs show parameters that represent the effectiveness of both methods: DR (left) and FIC (right). The lower graphs show comparative slow wave parameters between fasting and fed: SWF (left) and RMS (right). p value is indicated when there is a significant difference (p < 0.05) together with the sample size effect indicated with Cliff’s delta in brackets

Figure 3 shows the DFCS, FIC, SWF and RMS fast and fed box and whisker plots, with and without MVMD. Gastric SW detectability (DFCS) increased significantly after MVMD, in both fast and fed: from 49.3 ± 17.4 to 91.7 ± 5.6% in the fasting stage and from 59.7 ± 23.7 to 91.4 ± 8.4% in fed, with p values of 0.0002 for both comparisons and with very large (0.99) and large (0.78) effect sized respectively. No statistical differences were found in DFCS between fast and fed either with or without MVMD.

The FIC of the gastric SW diminishes significantly in fast and fed when identifying the fundamental mode of the gastric activity by MVMD, compared to not applying MVMD (raw signal): in fasting FIC is reduced significantly after MVMD, from 12.3 ± 2.7 to 5.3 ± 1.4% (p value: 0.0002) and from 10.3 ± 1.4 to 5.2 ± 2.0% postprandial (p value: 0.002) with very large (0.99 and 0.70) Cliff’s delta values. Statistical differences were not found in FIC between fasting and fed, either with or without MVMD.

Gastric SWF without MVMD was 2.80 ± 0.30 cpm in fasting, being slightly higher postprandially, 2.92 ± 0.36 cpm, without statistically significant differences. After MVMD, gastric SWF was 2.88 ± 0.24 cpm in fasting, with a statistically significant increase to 3.06 ± 0.19 cpm in fed. Gastric SWF values after MVMD were slightly higher than those without MVMD. Indeed, there was no significant difference for SWF either with or without MVMD in fasting but there was a statistically significant difference in fed with a p value of 0.003 and a moderate Cliff’s delta value (0.4).

Gastric SW RMS was noticeably higher after ingestion, both with and without MVMD: 91 ± 21 µV in fasting and 155 ± 77 µV in fed without MVMD (p value: 0.0002), and 33 ± 9 µV in fasting and 63 ± 30 µV (p value of 0.0005) in fed. As expected, a higher gastric SW amplitude was found without MVMD in both states because RMS values after MVMD were only those of the fundamental component of the gastric SW. Significant differences were found in SW RMS, without and with MVMD in fasting (p value: 0.0002) and fed (p value: 0.0002) with very large effect sizes of (0.81) and (0.84) respectively.

### Slow wave vectorgastrogram $(VGG_{SW}$)

#### Trajectory dynamics

**Fig. 4:**
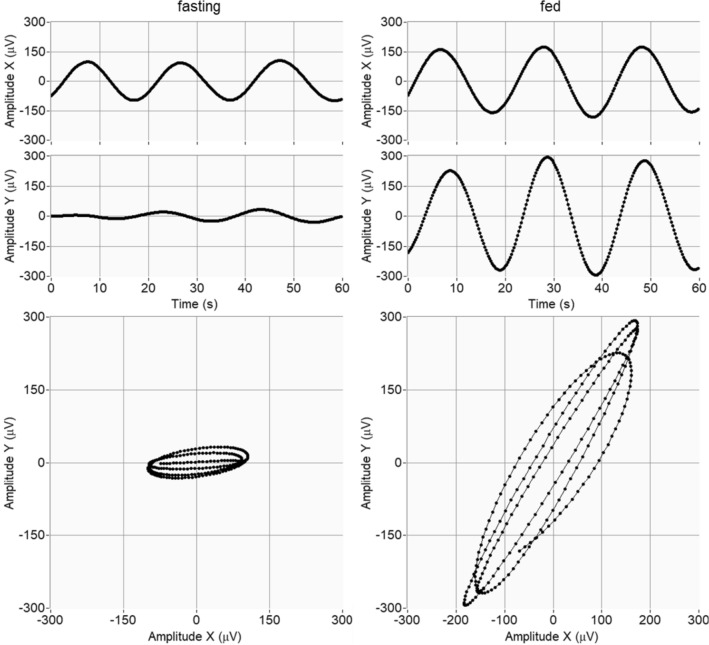
$\overrightarrow {{VGG_{SW} }}$ vectorgastrograms (bottom) for fasting (left) and fed states (right) calculated from the upper signals, which are bipolar combinations of channels 1–4 (X axis, upper plots) and bipolar combinations of Channels 3–18 (Y axis, middle plots). For clarity, the X and Y signals and the VGG were obtained for 60 s; i.e. around 3 SW cycles

**Fig. 5:**
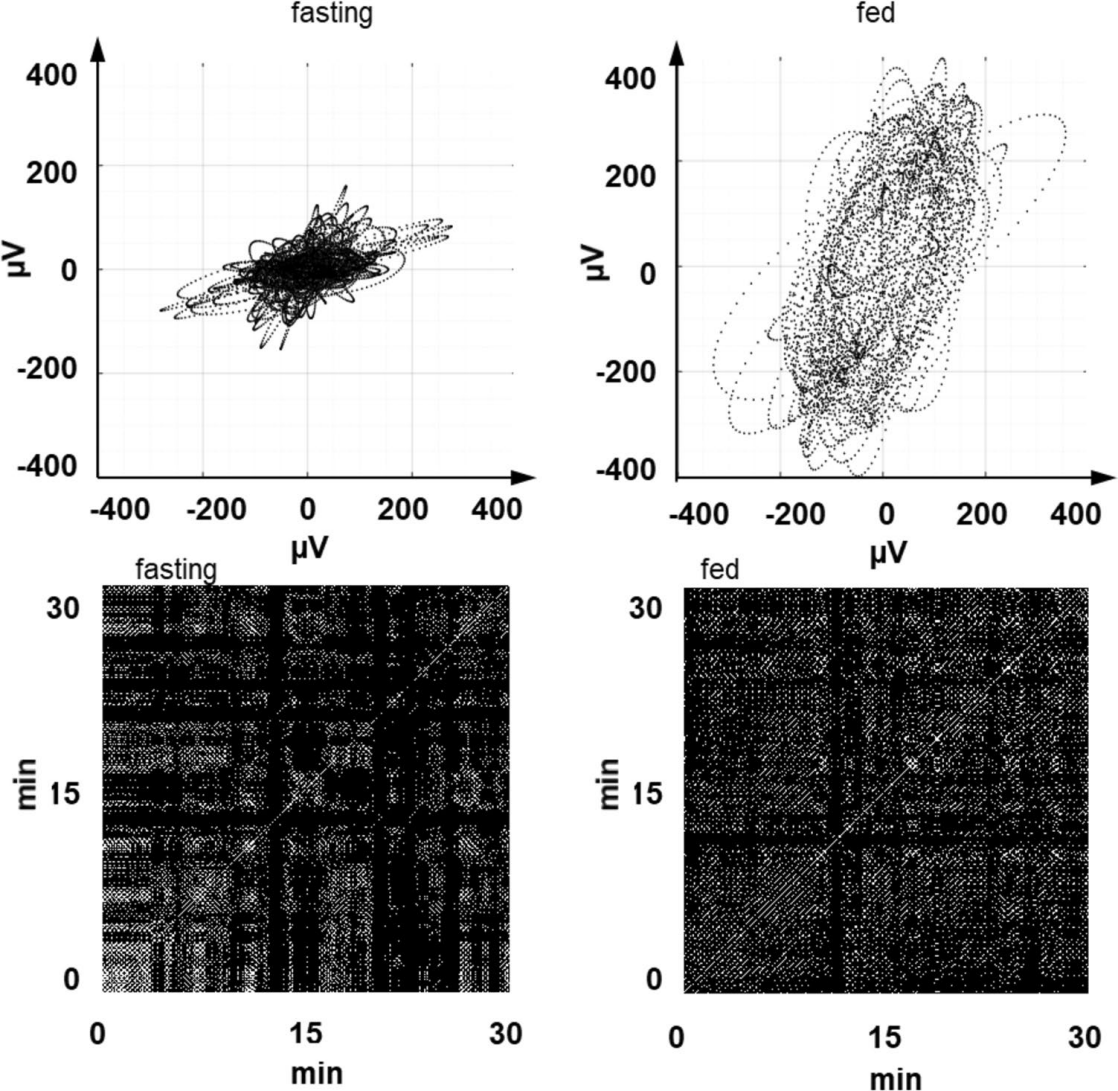
Vectorgastrograms of the 30-min recording (upper) and threshold (th = 0.4) recurrence matrix (lower) obtained from the above $\overrightarrow {{VGG_{SW} }}$ in Subject 1 in fasting (left) and fed state (right graphs)

As commented above in Materials and Methods, VGG_SW_ was computed on the fundamental modes obtained from the MVMD, since they offer greater robustness in slow wave capture. Figure 4 gives 60 s (three signal cycles) of the X (Channels 1–4) and Y (Channels 3–18) modes after MVMD from Subject 1 in fasting (left) and fed (right) and their corresponding VGG_SW_. Both the X and Y signals increased their amplitudes after ingestion, much more so in the Y signal. In both cases a frequency of about 3 cpm is can be seen. There is evidence of increased verticality of VGG_SW_ trajectory after ingestion. For the whole record, Fig. 5 depicts 30 min of the VGG_SW_ obtained from Subject 1 when fasting (upper left) and postprandially (upper right) and the corresponding recurrence matrixes. VGG_SW_. excursion in both the X and Y axes increased after ingestion, being more noticeable in the Y axis, as reflected in VGG_SW_.

The recurrence matrixes (see Fig. 5, lower traces) showed an increase in the number of recurrent dots (black after ingestion, which indicates enhanced predictability after ingestion, resulting in greater RR, also in the number of dots that form diagonal lines (parallel to the main diagonal) over the total dots, revealing a more deterministic structure (increased fed DET. A slightly greater average length of the diagonal lines can also be seen, indicating a more constant trajectory after food.

Box and whisker plots of VGG_SW_ parameters and those from the recurrence matrix in fasting and fed states are shown in Fig. 5. R1 increased significantly after ingestion, from 1.09 ± 0.41 to 1.58 ± 0.87 (p value: 0.04, Cliff’s delta 0.38), showing a rise in the accumulative differential absolute vertical displacement of the VGG_SW_. Higher values for R2 were also obtained after ingestion (from 9.36 ± 5.14 to 12.29 ± 9.04) but with no significant differences between fasting and fed. Rv2 values were 1.81 ± 3.32 in fasting and significantly higher in fed (3.12 ± 2.6) (p value: 0.03, Cliff’s delta 0.42), i.e. a greater maximum slope of the trajectory described by the VGG_SW_ after ingestion. The angle rose from 224.8° ± 29.58° in fasting to 245.4° ± 25.8° in fed, with greater verticality) (p value: 0.004, Cliff’s delta 0.40). In agreement with the previous results, there was a decrease in the ratio between the major and the minor axes (AXR), identified by principal component analysis (PCA), significantly dropping from 1.97 ± 0.22 in fasting to 2.19 ± 0.24 in fed (p value: 0.006, Cliff’s delta 0.48).

#### Recurrence quantification analysis (RQA)

Regarding the RQA metrics, RR, DET, LMEAN, ENT and TT increased after ingestion, with statistically significant differences between fasting and fed, except for DET. RR is the RQA metric, which presented the most significant difference between fasting and post-ingestion, the lowest p value, increasing from 22.48 ± 1.1 in fasting to 24.4 ± 2.04 (p value: 0.007) in fed, indicating higher VGG_SW_ predictability, which is consistent with the significant increase in the LMEAN, rising from a fasting value of 0.73 ± 0.12 to 0.94 ± 0.23 in fed (p value: 0.017). ENT was the second RQA metric with the lowest p value in discriminating between fasting and fed states (5.99 ± 0.32 in fasting to 6.36 ± 0.4 in fed, p value 0.010). In contrast to the classical entropy metrics, which raise their values when signal complexity and chaos increase, the entropy obtained from a recurrence plot is linked to the inverse of the largest Lyapunov exponent and thus decreases when signal complexity and chaos increase. It can thus be interpreted that the VGG_SW_ degree of complexity significantly decreases after ingestion. Finally, the TT parameter points to a significant increase in VGG_SW_ stability after fed (0.47 ± 0.04 in fasting and 0.51 ± 0.06 in fed, p value: 0.02). These values are around half the period of a slow wave, which is approximately 10 s (slightly lower than 10 s fasting and slightly higher than 10 s postprandially).

**Fig. 6:**
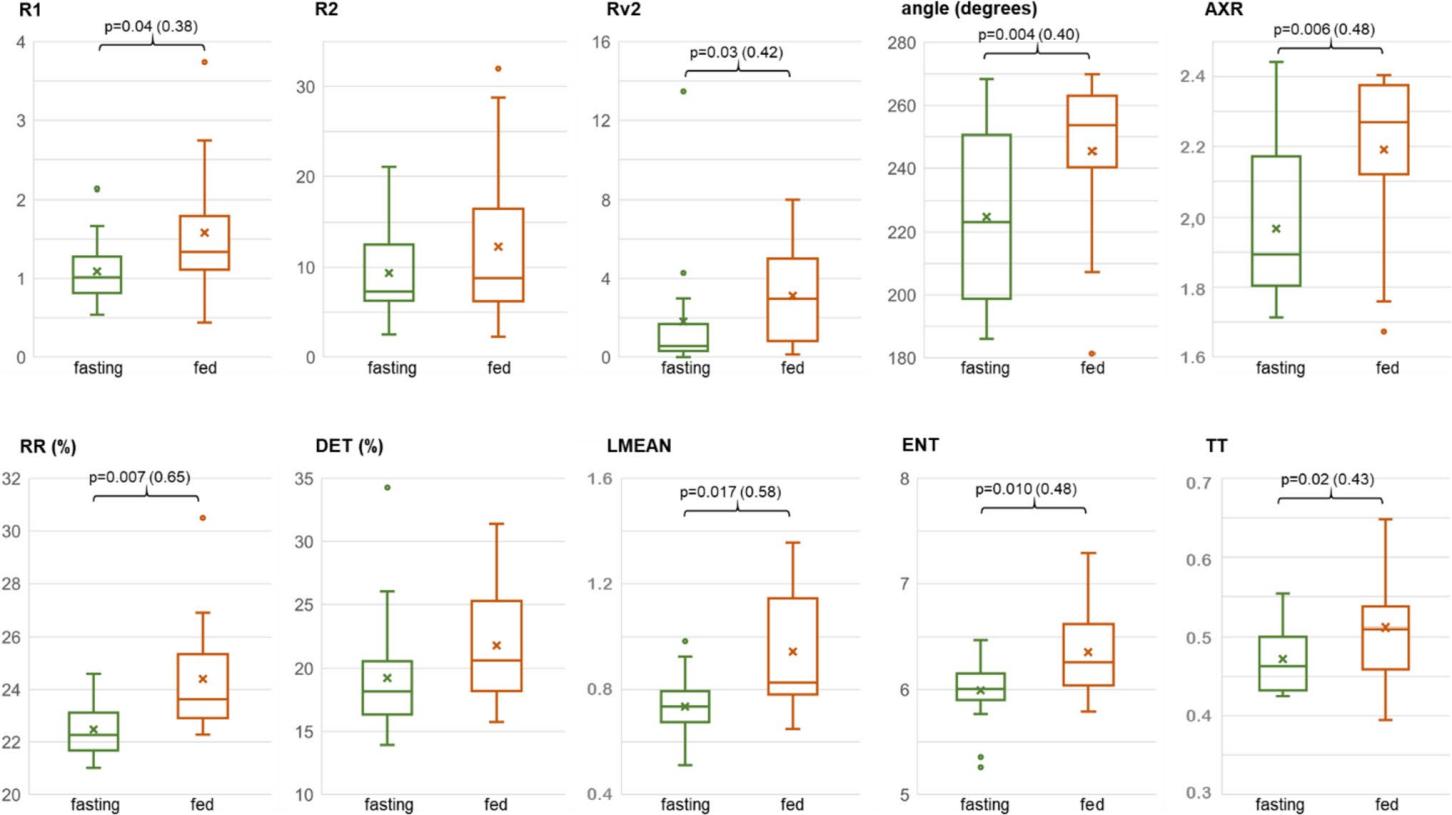
Box and whisker plots of the parameters calculated from VGG (upper graphs) and parameters from recurrence matrix (lower graphs), comparing fasting (green plots) and fed states (orange plots). p value is indicated when there is a significant difference (p < 0.05) together with the sample size effect indicated with Cliff’s delta in brackets

RQA metrics with significant p values < 0.05 (RR, LMEAN, ENT and TT) were associated to slightly higher Cliff’s delta values (from 0.43 to 0.65), than those corresponding to trajectory dynamics (from 0.38 to 0.48). These results remark on the relevance of the use of these biomarkers for detecting SW changes (Fig. 6).

## Discussion

Pathological conditions or circumstantial causes can alter gastric activity and the changes can lead to gastric dysrhythmia (bradygastria or tachygastria), abnormal slow wave propagation or electromechanical uncoupling [59]. The main stumbling block that has prevented electrogastrography from being transferred to clinical practice has been the difficulty of obtaining robust metrics associated with the frequency, amplitude and/or direction and speed of propagation of the gastric slow waves, which continues to be a challenge. This can be attributed to the gastric SW frequency spectrum being close to or even overlapping those of other types of interference, such as the skin–electrode contact potential. The frequency of the gastric SW has traditionally been associated with the dominant frequency of the filtered EGG signal in the gastric SW bandwidth.

Previous studies have proposed using the dominant frequency in the multichannel EGG recording cross-spectrum (DFCS) for robust assessment of high spatial stability components [46, 60] or applying empirical mode decomposition to detect intrinsic mode functions [20, 61]. Other works use a second order blind identification (SOBI) technique for this purpose, especially in magnetogastrographic [62] and manetoenterographic recordings [63]. In the present work, robust multichannel identification of gastric activity was carried out non-invasively by a 5 × 5 electrode array positioned over the abdomen and applying the multivariate variational mode decomposition technique (MVMD) in the fasting and fed states. The MVMD approach overcomes the deficiencies of the multivariate extension of empirical mode decomposition, such as the low effectiveness of low sample levels and artifacts, a shortage of mathematical formulations and mode mixing, of several channels [64]. MVMD tends to be more robust in the presence of noise and artifacts than SOBI approach and performs[44] well when the number of employed channels is limited, and its performance does not worsen with an increase in channels [48].

MVMD does not require predefined wavelet filter bank limits, which limits the multivariate extension of the empirical wavelet transform [63]. It also provides the mode separability feature, which is the disadvantage of the multivariate extension of the synchrosqueezing transform [64]. MVMD has previously been applied to enhance signal conditioning in EEG signals. Sharma and Singh [65] conducted a comprehensive experimental analysis to incorporate multivariate empirical-basis decomposition approaches in classifying the attention-deficit hyperactivity disorder in children. Sadiq et al. proposed using MVMD to identify motor imagery electroencephalographic (EEG) signals. The combination of MVMD from an 18-channel EEG in the motor cortex area achieved an average classification accuracy of 99.8% for a subject-dependent experiment, while the figure was 98.3% for subject-independent experiments, resulting in a flexible framework for subject-dependent or independent brain computer interface systems [64].

As far as we are aware, this is the first study to apply the MVMD technique to multichannel EGG signals. The results revealed that this technique improved identification of the gastric slow wave as compared to traditional processing techniques. The detection rate in DFCS rose by up to 91% when using MVMD and the frequency instability coefficient FIC decreased by up to 5.20% in fasting and fed after MVMD, these last values being lower [66] or similar to those obtained from bipolar EGG recordings in the fasting and fed stages in healthy volunteers in the literature [46, 67]. These results suggest that after MVMD it is not necessary to compute the cross spectrum to robustly identify the gastric slow wave, which greatly simplifies its identification. It should be noted that DFCS without MVMD was lower than those obtained in other works in the literature for EGG bipolar recordings (> 80%) [46, 66]. This can be attributed to those bipolar EGG recordings analysing monopolar recordings, which are more susceptible to interference, such as skin–electrode, contact than bipolar recordings. It should also be remembered that we used the mean value of DFCS of the entire electrode matrix, while the recording protocols used in the literature usually locate a smaller number of electrodes in the optimal position to pick up gastric activity [46, 59].

As for the gastric SW features, their frequency and amplitude increased after ingesting food, in agreement with previous works that recorded a rise of between 1 to 3 times for the gastric SW amplitude in the fed state [47]. The impact of eating on EGG amplitude can be ascribed to factors such as gastric distension, gastric displacement, stimulation of gastric contraction [68], and alterations in gastric slow waves due to the presence of spike bursts [69, 70]. In a previous work of our research group [46], we also confirmed that the amplitude of the bipolar Electrogastrogram (EGG) and the power ratio (fed/fasting) increased after solid meal ingestion. Furthermore, in healthy volunteers, the consumption of solid food with a calorie content of up to 400 kcal and less than 50% fat was demonstrated to increase both the amplitude and frequency of the gastric slow wave (Kasicka-Jonderko et al., 2006). This is in accordance with the results in the present work and similar studies [46, 47]. By contrast, literature also observed a reduction in gastric SW frequency after liquid ingestion [47, 67]. In the present work the increase in frequency after solid meal ingestion, 400 kcal with a fat content of 18% and 0.25 L of water, was statistically significant only for the fundamental component of the gastric SW, identified after MVMD, changing from 2.88 cpm in fasting to 3.06 cpm postprandially, as observed in the literature [46, 47, 71, 72]. In healthy volunteers, the consumption of solid food with a calorie content of up to 400 kcal and less than 50% fat was demonstrated to enhance both the amplitude and frequency of the gastric slow wave [71]. Physiologically, the intake of high-fat meals would activate the function of the vagus nerve, sending a signal to the brain that produces a feeling of satiety and the desire to stop eating. It has been proven that vagal nerve stimulation, which activates enteric motor neurons, increases the frequency of slow-wave activity in the gastric antrum [73]. The increase in frequency and the ability to pace slow waves through neural stimulation are mediated through interstitial cells of Cajal (ICC) [74]. In addition, high-fat feeding, which causes systemic inflammation and disrupts the interbalance of enteric innervation, enhances gastric smooth muscle contractility [75]. In this regard, Chen et al. showed that the slow wave frequency correlates well with stomach contractions by means of simultaneous recording of surface EGG and manometry in the stomach [76]. It has been also evidenced that gastric SW frequency increase is influenced by the mechanotransduction mechanism triggered by gastric overdistension: stretching the ICC-IM led to a membrane depolarization and a rise in gastric slow-wave frequency [73].

It is noteworthy to mention that without MVMD, the fasted and fed SW amplitudes were higher than those reported in the literature for gastric SW from bipolar recordings [46, 77]. The greater susceptibility of monopolar recordings can explain this interference, such as the variation of the contact potential between the electrode and the skin. In contrast, the amplitudes of the fundamental components of the gastric SW identified after MVMD were similar to the gastric slow wave amplitude (GSWA) from bipolar EGG recordings [46]. Although a higher value for the former could be expected due to its monopolar origin, the similarity may be due to the GSWA in [46] being worked out from bipolar EGG recordings after being bandpass filtered between 0.6 and 9 cpm. Conversely, after MVMD, we selected only the mode with a dominant frequency between 2 and 4 cpm, i.e. the fundamental spectral component of the gastric SW. The amplitude of the fundamental component of the gastric SW (mode selected after MVMD) also increased postprandially, in agreement with the literature food [47].

The vector associated with the gastric SW was calculated and characterised in the fasting and fed stages. In the literature, only one publication from the early 1970s obtained the VGG_SW_ from a single thin healthy subject without conclusive results [27]. We computed the VGG_SW_ in healthy volunteers to assess the potential of this simple technique to provide helpful information on the EGG SW propagation patterns, paving the way to using electrogastrography in clinical practice. In the present work, the metrics that assess the vertical vs horizontal, total and global, displacement for VGG_SW_ (R1 and R2) the one that estimates the maximum slope of its trayectory (Rv2), and the angle, statistically increased after fed in healthy volunteers. These parameters have a low computational cost and their trends indicate the predominance of the VGG_SW_ vertical propagation direction after ingestion (R1 and R2) together with an increased vertical slope of the trajectory (Rv2). This could be due to the gastric motility after ingestion and the presence of the peristaltic contraction waves, originating in the proximal region of the gastric body with a frequency of 3 to 4 cpm, which are weak at first but propagate distally and progressively, increasing in intensity and reaching a maximum in the antrum. The stomach changes its shape (reduced volume) due to gastric motility [78], which may favour the VGG_SW_ higher vertical direction of propagation postprandially. An increase in the vertical orientation of the angle in fed healthy subjects was also observed. This reinforces the evidence of a more vertical direction propagation for the VGG_SW_ after ingestion. It should be noted that the values obtained for the angle in healthy fasting subjects are similar to those computed from the mean local propagation angles of the gastric slow waves [11, 21, 77]. To non-invasively estimate the direction and propagation speed of the gastric SW up to now the literature resorted to HR-EGG, obtaining average estimators of the SW speed and propagation direction calculated from “local” EGG recordings [11, 21]. High-density gastric mapping is cumbersome, time and resource-consuming and therefore difficult to introduce into routine clinical practice. We studied the gastric vector of the SW VGG_SW_ as an alternative technique for the global evaluation of the gastric slow wave propagation: VGG_SW_ assesses changes in instantaneous gastric vector displacement viewed from a longer distance, associated with propagated events rather than local activity [28]. A fundamental advantage for VGG_SW_ clinical use over high-density EGG mapping is that it can be obtained by placing only a few recording electrodes, which would be cheap and easy to use in clinics.

RQA metrics have been widely used to characterise vectorcardiographic signals [36–39, 42] and to discriminate imminent labour and non-imminent cases by means of electrohysterographic signals [79]. However, this is the first work in gastroenterology on recurrence quantification analysis by VGG_SW_. All RQA metrics increased significantly after ingestion, except for DET, which also showed an upward trend. DET and LMEAN, which quantify diagonal lines, increased after ingestion, indicating that the VGG_SW_ displacement increases its periodicity postprandially [40]. In addition, the higher values of TT after ingestion suggest that slow waves increase in stability. The Shannon entropy values are also higher in fed, which can be interpreted as less chaos [56–58]. The fed gastric SW electrical activity therefore becomes more intense and coordinated, with a less complex SW vector and with higher periodicity.

This supports the case for VGG_SW_ as a simple technique that can provide relevant information regarding the “global” spatial pattern of gastric slow wave propagation, thus being helpful for the diagnosis of gastric pathologies. In future work, we aim to increase the EGG database of healthy subjects, expanding the age and body mass index ranges and, even more critical, to obtain EGG recordings of pathological situations such as gastroparesis and chronic nausea. This will permit us to assess whether the information provided by the changes in the gastric SW instantaneous vector, VGG_SW_, such as direction, velocity, stability and complexity quantified by RQA are sensitive to the identification and/or follow-up of gastric pathologies such as gastroparesis, functional dyspepsia or reflux. We also aim to compute the VGG in the whole EGG bandwidth and assess its capacity for reflecting high-frequency electrical activity (spike bursts) associated with gastric motility, which will help to fine-tune robust EGG tools to aid in diagnosing gastric disorders.

## Conclusions

MVMD can robustly identify the fundamental component of the gastric SW, with detection rates over 91% in fasting and fed subjects with a frequency instability below 5.3%. The fundamental SW component identified after MVMD statistically increases its amplitude and frequency after ingestion. We also confirmed the feasibility of assessing slow wave vector movement by 2D-VGG in healthy subjects. The VGG trajectory dynamics revealed a statistically significant increase in vertical vs. horizontal displacement in the VGG_SW_ trajectory (R1 and R2 metrics) after ingestion. We also found a statistically significant increase in the verticality of the trajectory slope (Rv2) and fed VGG_SW_ angle, which highlights the predominance of the VGG_SW_ vertical propagation direction after ingestion. The RQA metrics (RR, LMEAN, ENT and TT) rose significantly after ingestion, except for DET, which also showed an upward trend. Gastric SW electrical activity after ingestion therefore becomes more intense and coordinated with a less complex SW vector and higher periodicity. These findings support the VGG_SW_ as a simple technique that can provide relevant information on the “global” spatial pattern of gastric slow wave propagation, thus being helpful for the diagnosis of gastric pathologies.

## Data Availability

Data can be available upon request to the authors.
